# Evaluation of Exome Sequencing to Estimate Tumor Burden in Plasma

**DOI:** 10.1371/journal.pone.0104417

**Published:** 2014-08-18

**Authors:** Daniel Klevebring, Mårten Neiman, Simon Sundling, Louise Eriksson, Eva Darai Ramqvist, Fuat Celebioglu, Kamila Czene, Per Hall, Lars Egevad, Henrik Grönberg, Johan Lindberg

**Affiliations:** 1 Department of Medical Epidemiology and Biostatistics, Science for Life Laboratory, Karolinska Institutet, Stockholm, Sweden; 2 Department of Pathology and Cytology, Karolinska University Hospital, Stockholm, Sweden; 3 Department of Clinical Science and Education, Södersjukhuset, Stockholm, Sweden; 4 Department of Medical Epidemiology and Biostatistics, Karolinska Institutet, Stockholm, Sweden; University of Kentucky College of Medicine, United States of America

## Abstract

Accurate estimation of systemic tumor load from the blood of cancer patients has enormous potential. One avenue is to measure the presence of cell-free circulating tumor DNA in plasma. Various approaches have been investigated, predominantly covering hotspot mutations or customized, patient-specific assays. Therefore, we investigated the utility of using exome sequencing to monitor circulating tumor DNA levels through the detection of single nucleotide variants in plasma. Two technologies, claiming to offer efficient library preparation from nanogram levels of DNA, were evaluated. This allowed us to estimate the proportion of starting molecules measurable by sequence capture (<5%). As cell-free DNA is highly fragmented, we designed and provide software for efficient identification of PCR duplicates in single-end libraries with a varying size distribution. On average, this improved sequence coverage by 38% in comparison to standard tools. By exploiting the redundant information in PCR-duplicates the background noise was reduced to ∼1/35000. By applying our optimized analysis pipeline to a simulation analysis, we determined the current sensitivity limit to ∼1/2400, starting with 30 ng of cell-free DNA. Subsequently, circulating tumor DNA levels were assessed in seven breast- and one prostate cancer patient. One patient carried detectable levels of circulating tumor DNA, as verified by break-point specific PCR. These results demonstrate exome sequencing on cell-free DNA to be a powerful tool for disease monitoring of metastatic cancers. To enable a broad implementation in the diagnostic settings, the efficiency limitations of sequence capture and the inherent noise levels of the Illumina sequencing technology must be further improved.

## Introduction

All human individuals harbor cell-free DNA (cfDNA) in the circulation [1], [2]. In cancer patients, as tumor cells die, DNA is shed into bloodstream. Circulating tumor DNA (ctDNA) constitute a fingerprint, which can be used for disease monitoring. Circulating tumor DNA has been correlated to both early detection and prognosis [3], [4]. Since the half-life of cfDNA is less than one hour, it has been successfully used to monitor treatment progression [5], [6]. Although ctDNA is an extremely promising biomarker, clinical implementation has been impeded, not only by inherent challenges in the characteristics of cfDNA, but also in tumor biology as well as technology. Cell-free DNA is present in low concentration, and the majority of fragments are short which limits the efficiency of PCR based methodologies. Circulating tumor DNA fractions are low, except in metastatic and high-grade disease. Levels of ctDNA was demonstrated to be <1% on average for non-metastatic colorectal tumors [4] which marks the upper bound for a desired sensitivity. Furthermore, as revealed by the large ongoing cancer sequencing efforts, any two individuals harboring the same cancer diagnosis share few, if any, somatic events [7], which require a high degree of flexibility. Various methods have been used for the detection of tumor-specific somatic lesions in the circulation. Monitoring genomic break-points through digital PCR is highly specific, allowing for the detection of single copy cancer genomes in milliliters of plasma [5], [6]. Sequencing of a selected subset of genes has demonstrated potential to detect ctDNA down to 0.14% [8] and excellent correlation to orthogonal technologies such as digital PCR [9]. Chan and colleagues demonstrated the feasibility of using whole genome sequencing of plasma DNA in cancer patients to detect somatic copy number alterations according to the same rationale as previously shown for trisomy 21 [1], [10]. Although promising, the approach requires unfeasible deep coverage for a sensitivity level of 1% [11]. Recently, Murtaza and colleagues displayed the advent of exome sequencing to monitor multiple mutations in concert. Targeted sequencing still has the economical advantage over whole-genome sequencing, while capturing the majority of known driver mutations [7]. Nevertheless, these individuals suffered from metastatic late stage disease and whether the sensitivity is good enough for detection of low levels of ctDNA remains unknown [12]. Additionally, unlike other assays [13], whole-exome sequencing does not require individual assays to be tailored for the vast majority of patients [14], a requirement for a broader clinical utility. Here we investigate the utility of using exome sequencing for monitoring of ctDNA levels through detection of single nucleotide variants in plasma. Since the number of variants is commonly <100 for most solid tumors [14] and in order to retain maximal sensitivity we 1) evaluated the capability of two promising approaches to generate sequencing libraries with high complexity from small amounts of fragmented DNA without pre-amplification; 2) optimized data analysis pipelines for read depth and accuracy; 3) applied exome sequencing on plasma obtained from prostate cancer and breast cancer patients to demonstrate its utility. In conclusion, the main limiting factor was the low efficiency of library preparation and subsequent targeted capture. Less than five percent of starting molecules were observable, limiting the sensitivity to 1/2433 using 10 ml of plasma, thereby restricting the applicability to locally advanced cancers reported to emit fragments into the circulation.

## Materials and Methods

### Samples and clinical data

Prostate tumor tissue and clinical data were collected from men who underwent radical prostatectomy at the Karolinska University Hospital in Stockholm as described previously [15]. Blood was collected at patient registration in the eve of surgery, directly after surgery, at discharge and at return visit. Breast tumor tissue was collected from women who underwent surgery for breast cancer at the South General Hospital in Stockholm. Blood was collected at the patient registration, approximately one week prior to surgery. Signed informed consent was obtained for all study participants. The ethical approval was give by the Regional Ethical Vetting Board in Stockholm (located at the Karolinska Institutet) with registration numbers 2009/1357-32 (prostate cancer samples) and 2010/958-31/3 (breast cancer samples).

### Extraction of nucleotides

DNA was extracted from whole blood using QIAmp spin miniprep kit (Qiagen, Hilden, Germany). DNA/RNA and proteins were simultaneously extracted from prostate and breast cancer tissues, as described previously [15]. Cell-free circulating DNA was isolated from plasma using QIAamp Circulating Nucleic Acid Kit (Qiagen). All extractions were done according to the manufacturers recommendations. High molecular weight fragments were removed from the cell-free circulating DNA samples by polyethylene glycol (PEG) mediated precipitation on carboxylic acid coated magnetic beads (MyOne, Invitrogen) as described previously [16] using 8% and 25% PEG 6000 (Merck) in the first and second solution respectively in a Magnatrix™ 1200 (NorDiag ASA, Oslo, Norway) liquid handling robot. DNA concentrations were measured using a Qubit fluorometer (Invitrogen, CA, USA) dsDNA HS kit and the size distributions of the cell-free DNA were assessed using Agilent 2100 BioAnalyzer (Agilent Technologies, Santa Clara, CA, USA) and the DNA HS kit.

### Simulated ctDNA

DNA samples derived from tumor tissue was sheared by suspension in 120 ml nuclease free water and sonication using the Covaris (Covaris Inc., MA, USA) sonication system using the settings for a 150 bp peak according to the manufacturers instructions. 1 µl of each sample was analyzed using an Agilent 2100 Bionalyzer and the DNA 1000 kit. Automated size-selection was done as described previously [16] using 10% and 11% PEG 6000 (Merck) in the first and second solution respectively in a Magnatrix™ 1200 (NorDiag) liquid handling robot and the resulting size distributions were assessed using Bionalyzer and the DNA 1000 kit (figure S1).

### Exome capture

For the evaluation of performing exome sequencing on minute amounts of sample, sequencing library preparation was done from 1 and 10 ng DNA derived from prostate cancer tissue using Mondrian SP+ System (NuGEN Technologies Inc., CA, USA) or ThruPLEX-FD Prep Kit (Rubicon Genomics, MI, USA) according to the manufacture's recommendations. Exome capture was performed as described previously [15]. Custom blocking adapters were used for respective technology. For primary tumor material, whole blood of plasma, libraries were prepared ThruPLEX-FD Prep Kit (Rubicon Genomics). Exome capture was carried out using the SeqCap EZ Exome Library Version 1 (Roche Nimblegen Inc, Madison, WI, USA) according to the manufacturers instructions.

### Sequencing

Sequencing was carried using Illumina 2×100 bp paired-end sequencing on a HiSeq 2500 instrument according to the manufacturers recommendations using TruSeq PE Cluster Generation Kit v3 and the TruSeq SBS Kit v3. All lanes were spiked with 1% phiX as a quality control.

### Processing of sequence data

Three analysis pipelines were implemented to compare the performance of using 1) standard sequencing processing 2) standard sequencing processing but with merging overlapping paired-end reads to improve base qualities and reduce noise rates 3) standard sequencing processing but with merging overlapping paired-end reads with subsequent optimized PCR duplicate processing to improve base qualities and to reduce noise rates. Standard sequencing processing was defined as 1) removal of adapter sequences only, using SeqPrep (v. 1.1) [17] 2) alignment to the reference genome (hg19) using BWA (v. 0.6.2) [18] 3) realignment using GATK [19] 4) removal of technical duplicates using Picard [20]. All QC metrics were obtained using Picard. Sequence data from tumor tissues and normal blood DNA was processed using standard sequencing processing. Realignment and base quality recalibration was carried out using GATK v2.8-1 before the identification of somatic point mutations using Mutect v 1.1.5 [21]. Merging of overlapping reads was performed using the SeqPrep software. SeqPrep was modified to set discordant overlapping base pairs to N with quality 2. Concordant base pairs were used to boost base qualities by addition to a maximum of 45. The modified version of the SeqPrep is available at https://github.com/dakl/SeqPrep. For optimal utilization of data and to further improve noise rates MergeDuplicates was designed. Unlike MarkDuplicates, provided in the Picard software suite, MergeDuplicates takes amplicon length of single-end data into account for the identification of PCR duplicates and also merges duplicates, to provide a consensus call, for increasing base qualities (figure S2). For each set of duplicate molecules, each base is traversed and if at least 75% of bases in each position are identical, this base is kept and the phred-scaled qualities are boosted by addition, otherwise the base is set to N with quality 2. Maximum base quality was set to 50. MergeDuplicates is available at https://bitbucket.org/dakl/mergeduplicates. Note, the boosted base qualities from SeqPrep or MergeDuplicates were not used for variant identification but as a means of separating data with support from multiple independent sources at the level of overlapping sequencing (modified version of SeqPrep) and PCR duplicates (MergeDuplicates). Mutational data for each position was obtained using Samtools [22]. Samtools associates each base with a Base Alignment Quality (BAQ). The BAQ gives the phred-scaled probability of each base being misaligned and is the minimum between base quality and the BAQ [23]. To reduce background noise, variants were filtered to remove positions residing in regions of the human genome with low uniqueness (mappability) [24] or known to harbor germline variants (fig. S3). To further restrict to regions with excellent mapping characteristics, variants were not allowed if within 50 bp from a region with mappability <1. Data management and statistical analysis were done in R [25].

### Simulation

To investigate the sensitivity of using exome sequencing in plasma, the following variables were varied; proportion ctDNA (range 0.00001–0.05), amount of starting material (range 3–60 ng). As the quality of the background variant reads were lower relative to reference bases, a quality filter was set where the maximum fraction of reference reads was kept relative the noise. For simplicity an exome was assumed to contain 50 variants, which in concert with the determined assay efficiency set the collective depth for each iteration. Also, sample bases were drawn with a probability to draw a variant base equal to the current fraction of ctDNA. For the background, the whole set of data was used for each iteration. Subsequently a one-sided fishers exact test was performed to test if the fraction of ctDNA was significantly higher relative to the background data. This process was repeated 1000 times for each fraction of ctDNA and each amount of starting material.

### Identification and validation of somatic rearrangements

To identify somatic chromosomal rearrangements, we performed whole-genome sequencing using of long-insert (approximately 700 bps) libraries for the breast tumors and paired normal DNA from blood. DNA was fragmented using a Covaris S1 system with the following settings: Duty Cycle 5%, Intensity 3, Cycles per burst 200, and Time 50 s. Fragmented DNA was prepared as described previously [26] and sequenced to an average of 3× base coverage on an Illumina HiSeq 2000 system. From the WGS data, we used BreakDancer 1.3.5.1 to identify candidate somatic break-points and BICseq to identify copy number variants. We manually filtered these data to keep regions with good support from breakdancer as well as CNV support from BicSeq. To generate primer pairs for validation, reads spanning the breakpoints were extracted from the original fastq files. Each read pair was concatenated (read 2 reverse complemented) with a 30N spaces between them and fed into primer3 for design. In order to minimize the risk that sequencing errors were used in the primer design step, primer3 was instructed not to allow any bases with a quality <20 in the primers. For each breakpoint, the highest scoring primer pair was used. In total across 5 patients (BC_B, BC_C, BC_D, BC_F, BC_G), 19 primer pairs were design, out of which 18 validated giving a band specific to the tumor. For the primer pairs that gave unspecific product, the shortest band was specific to the tumor. Sanger sequencing confirmed the 18 rearrangements. We selected 8 of the rearrangements for analysis in the plasma samples (B3, C1, D1, D3, F4, F5, G1, G2). All rearrangements except two gave good signals in the tumors, with estimated allele frequencies between 5% and 78%.

## Results

### Evaluation of library preparation methodologies

**Figure 1:**
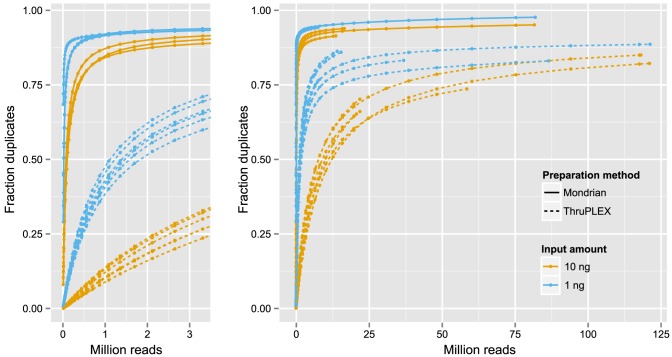
Duplication rates using the ThruPLEX kit and the Mondrian system. The proportion PCR duplicates in relation to sequencing depth demonstrated by subsampling of deeply sequenced libraries. **A**) The lower range and **B**) the higher range of sequencing depth. At any given number of reads sequenced, libraries with an input amount of 10 ng shows a lower fraction of duplicated reads compared to 1 ng. Furthermore, ThruPLEX-prepared libraries consistently show lower fraction of duplicated reads compared to Mondrian-prepared libraries.

A key aspect of performing exome sequencing of cfDNA is efficient library preparation as 1 ml of plasma from prostate cancer (PC) or breast cancer (BC) patients yields commonly yields 3 ng of cfDNA (data not shown). To avoid amplification biases [27] we set out to evaluate candidate technologies claiming to enable sequence analysis of nano-gram levels of DNA. As cfDNA is heavily fragmented (∼180 bp peak), the tagmentation-based kit from Illumina (Nextera) was excluded as it causes further shearing of the template DNA. To obtain enough DNA for repeated comparisons and to create a source of simulated cfDNA (figure S1), DNA from a tumor, previously profiled using whole-exome sequencing (SWE-54) [15] was carefully prepared to mimic the true size distribution of cfDNA. The simulated cfDNA was prepared for capture and sequencing using the ThruPLEX kit (Rubicon Genomics) and the Mondrian system (NuGEN Technologies). To optimize procedure efficiency, we evaluated: 1) amount of starting material (1 and 10 ng); 2) number of cycles of PCR performed after capture (9 and 18 cycles); 3) the capture plexity (1, 4 and 8 plex capture, figure S4). Capture was performed using a 5 Mb kit (∼1300 genes associated to cancer) to facilitate sequencing to saturation, and thereby, to better assess the limitations of respective technology. Assay performance was investigated using tools in the Picard software suit [20]. To ensure a successful capture using both technologies, the fold enrichment of target regions was assessed using only 10.000 reads, a level of sequence depth where complexity is not limiting (Mondrian range 390–440 fold enrichment, ThruPLEX range 370–400 fold enrichment). Library complexity was estimated as the average sequence coverage obtained after removing PCR duplicates. To enable a comparison of complexity throughout the whole range of sequence depths, the data was subsampled, starting with 10.000 reads, incrementing with 1.25× until all available data was used for each sample (figure 1). Furthermore, analysis of variance was performed (table S1) to estimate the significance of each factor, which revealed only the starting amount of DNA and the library preparation approach to be relevant for library complexity. The average coverage using 1 ng and 10 ng of starting DNA was 2x and 3x for the Mondrian system and 16x and 85x using ThruPLEX, respectively. Therefore, we used the ThruPLEX technology for further processing of plasma samples.

### Optimized procedures for exome sequencing of cell-free DNA

To evaluate the possibility of tracking point mutations in plasma we performed whole-exome sequencing on tumor tissue and blood from seven BC and one PC patient (table 1). This identified somatic variants for each individual, with the potential to act as personalized biomarkers in the circulation. Subsequently, we performed exome sequencing on plasma samples obtained before (all patients) and 1 month after surgery (the PC patient only). The level of uniqueness varies throughout genomes as do alignment-related errors, even in the presence of stringent filtering [28]. Therefore, to test if the fraction of variant reads found in cfDNA is significant relative to noise, the same positions must be used in the background distribution of samples. As background for each position we used data from all plasma samples profiled here, excluding samples where identical mutations were found in the primary tumor material. To obtain a high-quality call set, all variants identified in the primary tumor tissue were restricted to unique regions, not previously reported to harbor SNPs (figure S3). This removed 24% of all positions, retaining on average 81 variants per individual.

**Table 1:** Clinical and profiling data.

		Tissue/Blood exome sequencing			Plasma DNA exome sequencing					dPCR breakpoint profiling	
SampleID	Clincal data	Tumor*	Blood*	Nbr point mutations	cfDNA source	DNA (ng)	Plasma*	Fraction ctDNA	P-value	DNA (ng)	Fraction ctDNA
SWE-54_B	Gleason 5+4, T3A	87	86	27**	Before surgery	3	39	0.001	0.265	NA	NA
SWE-54_A	Gleason 5+4, T3A	87	86	27**	1 month after surgery	8	42	0	1	NA	NA
BC_A	Elston 3, prolif 75%, 21 mm, ER+, PR+, HER2+	122	147	26	Before surgery	5	62	0	1	5	NA
BC_B	Elston 3, prolif 70%, 18 mm, ER+, HER2+	114	137	184	Before surgery	1	18	0	1	1	0
BC_C	Elston 2, prolif 13%, 16 mm, ER+, PR+	111	146	17	Before surgery	5	57	0	1	5	0
BC_D	Elston 3, prolif 90%, 18 mm, ER+, HER2+	101	118	245	Before surgery	3	43	0.003	1.16E-18	3	0.026
BC_E	Elston 1, prolif 10%, 38 mm, ER+, PR+	134	188	20	Before surgery	5	55	0	1	5	NA
BC_F	Elston 2, prolif 2%, 12 mm, ER+, PR+	175	153	82	Before surgery	5	48	0	1	5	0
BC_G	Elston 3, prolif 85%, 24 mm, ER+, PR+	163	168	47	Before surgery	5	39	0	1	5	0

*Mean coverage throughout the exome.

**The point mutations originate from the two lymph-node metastases that were sequenced in ref 15.

A potentially limiting factor of sensitivity is the overall error rate of the Illumina sequencing technology, which was recently reported to be as high as 0.38% in cell-free DNA [10], here found to be 0.29%. Previously, and as Illumina offers paired-end sequencing, overlapping sequencing have been used to reduce errors made during sequencing by synthesis [29]. As cfDNA is fragmented (∼180 bp) we explored this option, although this does not allow for the correction of PCR errors occurring before sequencing. Commonly, during low-level processing of sequence data, PCR duplicates are identified through the mapped starting positions of individual reads. As these duplicates originate from the same molecule, they offer means to identify PCR errors and to decrease the error rate. Therefore, we explored the variation in noise rates, going from standard sequencing processing to merging of overlapping reads and lastly, using PCR duplicates to reduce noise (analysis pipelines 1–3, methods). Additionally, for paired-end data, the mapped position of both ends is used for efficient identification of PCR duplicates. In contrast, only the starting position is used for single-end data, as all fragments commonly have the same read-length. For cfDNA, and for other merged libraries with short insert-sizes, the distribution of fragment sizes obtained after merging offers means to distinguish reads originating from different starting molecules harboring the same mapped starting position (figure S2). On average, not merging reads caused an average inflated coverage of 58% relative merging and using standard MarkDuplicates provided in Picard [20]. Taking fragment size into account substantially improved coverage. There was only a 15% difference between not merging and merging in combination with MergeDuplicates (custom software, figure S5).

**Figure 2:**
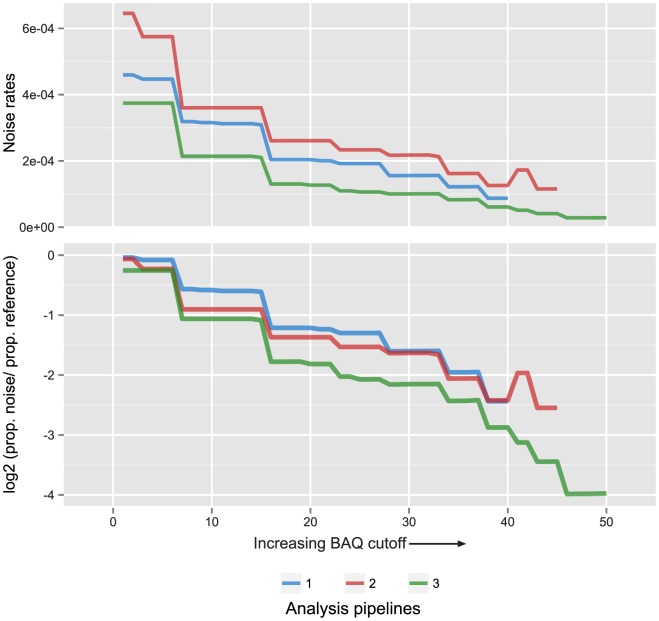
Base alignment quality filtering to reduce noise. **A**) The noise rate in background samples for analysis pipelines 1)–3) as the base alignment quality (BAQ) cutoff is increased. Rate is defined here as total number of mutant reads/(total number of mutant reads+the total number of reference reads) in the background samples at mutated positions. **B**) The log2 ratio of (proportion of mutant reads)/(proportion of reference reads) left by increasing BAQ cutoffs in the background samples at mutated positions. Colors scale according to analysis pipelines. Pipeline 1) BAQ limited to 40, as qualities were not altered. Pipeline 2) BAQ limited to 45 through merging of overlapping reads. Pipeline 3) BAQ limited to 50 by merging reads and also accounting for concordance between PCR duplicates originating form the same starting molecule.

To minimize the background noise, an increasing BAQ (base alignment quality) filter was applied which demonstrated a significant decrease with increasing stringency (figure 2A). Proportionally, the highest fraction of reference reads relative the noise was retained at the minimum noise rate (figure 2B, table 2). Noise rate was defined as the number of reads supporting the presence of a mutation in the background samples divided by the total number of reference reads in the same positions. Merging reads in combination with optimal PCR duplicate processing yielded the lowest noise rate (1/35419) at a BAQ cutoff of 46 (table 2). Therefore, we processed all plasma samples according to pipeline 3) using a BAQ cutoff of 46.

**Table 2:** Comparison of analysis pipelines 1–3.

Pipeline^1^	Proportion of N-bases^2^	Optimal BAQ cutoff^3^	Proportion of data left^4^	Noise rate^5^	Sensitivity^6^
**1**	0.00018	No cutoff	1.00	1/2176	1/852
1	0.00018	38	0.40	1/11451	1/1372
2	0.00067	43	0.17	1/8673	1/775
3	0.00330	46	0.61	1/35419	1/2433

1) Analysis pipelines as described in Material and Methods.

2) Proportion of bases set to “N” during processing.

3) Optimal base alignment quality cutoff (BAQ).

4) Proportion of data left using the BAQ cutoff set in^3^.

5) Noise rate defined as the number of mutant bases in the background divided by the number of reference bases.

6) Sensitivity of exome sequencing to detect ctDNA based on in silico evaluation.

Finally, a one-sided Fisher's Exact Test was performed to assess if ctDNA could be detected in the patients' paired plasma samples (table 1, table S2). The prostate patient carried lymph-node metastases at surgery. The metastases were, in conjunction with the primary tumor, exome sequenced previously (SWE-54 A-C) [15] and variants from the metastases were used for ctDNA estimation. For this individual, the pre-surgical sample was positive for ctDNA, albeit non-significant, whereas the post-surgical sample was negative. For the breast cancer samples, only one of the six patients was positive (BC_D). The breast tumors sequenced here comes from a prospective collection of patients, consisting of newly diagnosed invasive breast cancers at least 1 cm in size. Interestingly, the only positive sample was also the one with highest proliferation (as determined by percent cells staining with Ki67, table 1). For validation purposes we performed whole-genome long-insert sequencing (700 bps inserts) of five BC tumors and paired normal samples to ≈3x base coverage, corresponding to approximately 10x physical coverage of the genome. This data was used to identify candidate breakpoints of somatic chromosomal rearrangements. In total, 18 of 19 candidates were validated using Sanger sequencing. A digital PCR assay was set up to for each break-point, using 3–15 ng of cfDNA. This verified BC_D to harbor detectable levels of ctDNA, whereas all others were negative.

### Sensitivity of whole-exome sequencing of cell-free DNA

Several factors affect the sensitivity, the background noise rate, the amount of cfDNA obtained from plasma, the fraction of ctDNA but also the efficiency of the sequence capture procedure, including library preparation and enrichment. Six of the independent library preparations performed, using the simulated cfDNA, were sequenced to such depth that the proportion of new unique molecules outside target regions was in majority (average duplicate rate, 81%). As we started with 1 or 10 ng DNA and based on the coverage retrieved, the fraction of starting molecules accountable for was 4.7%. Averaging over the whole set of samples sequenced here, also including lower duplicate levels, the average fraction was 3.8%.

**Figure 3:**
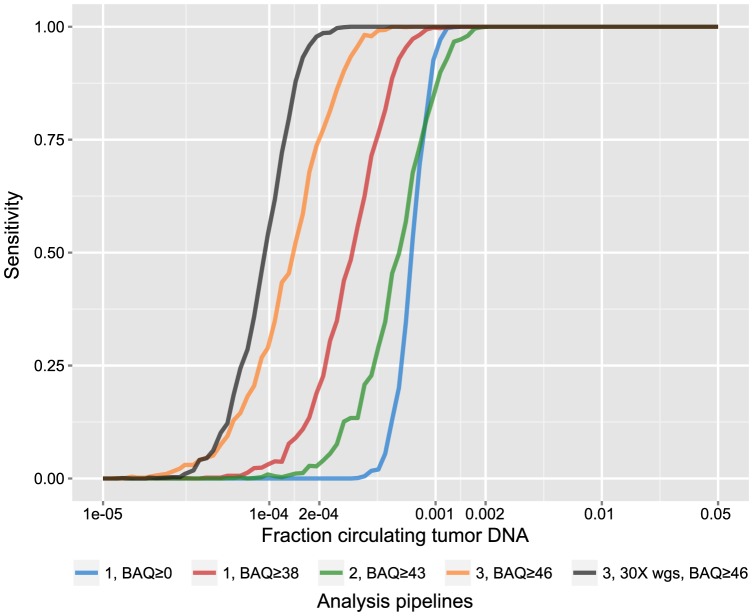
Sensitivity of exome sequencing to track ctDNA. Analysis pipelines 1)–3) are displayed here with optimal base quality alignment cutoffs (BAQ) and without for pipeline 1 to display the effects of BAQ filtering. As exome sequencing is limited by the efficiency of the capture procedure, 30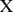 whole genome sequencing was also simulated assuming 3000 variants in the genome. 1000 iterations were performed for each ctDNA fraction assuming 50 variants in the exome, starting with 10 ml of plasma (30 ng of cfDNA). The sensitivity is defined as the number of proportion of tests passing the significance threshold for each set of 1000 iterations (p<0.05, fishers' exact test, comparing the number of variant and reference reads from sample and background).

To investigate how efficiency, preprocessing and other factors impact the sensitivity we performed an in silico evaluation. Since the simulated cfDNA libraries, used for technical evaluation, originated from a previously exome sequenced prostate tumor, variants were recalled (18 point mutations passing filters). Positions harboring mutations were used to sample variant- and reference reads in various fractions and depths representing the sample signal (9.4% mutation-supporting reads among all 4733 reads from 18 positions). The background/noise level was estimated from all plasma samples assayed here, collectively investigating all known variant positions in all tumor samples (678 positions), excluding each samples' own mutations, which accumulated 517973 reads for the simulation. Due to the low efficiency and to enable sensitive detection of ctDNA, all variant position were pooled and collectively tested vs. the background distribution of reads using a frequency test. Importantly, there was no difference in error rate (Wilcoxon rank sum test, p-value = 0.917) or size distribution (figure S1) between the simulated cfDNA and the real plasma DNA, a prerequisite to avoid inflation of BAQ:s. Several lessons could be drawn from this exercise (Figure 3); Due to the small proportion of data left after BAQ filtering (table 2) and in relation to ctDNA levels, boosting qualities through merging reads did not improve sensitivity. Improved processing of duplicates in combination with BAQ filtering gave the most sensitive approach, although limited to 1/2433 at 95% sensitivity. To reach such detection levels, 30 ng of cell-free DNA, commonly retrieved from 10 ml of plasma, is required. Further increasing input amounts by a factor of two, only lowered the sensitivity marginally due to the efficiency limitations of sequence capture (figure S6). As sequencing costs continue to drop, we investigated the sensitivity increase by whole genome sequencing (WGS) assuming 3000 variants per tumor genome and 30× average coverage. Although sensitivity was improved (1/5747) it is still not at the levels required to estimate the tumor burden in patients with locally confined, early stage tumors [13]

## Discussion

The presence of tumor fragments in the circulation holds promise to revolutionize care by offering efficient means to monitor systemic disease. This has spurred an active field of research, where many different approaches have been taken to assess ctDNA levels. Nevertheless, in order to become clinical routine, several requirements have to be fulfilled, including no only high sensitivity, but also practical applicability. Therefore we set out to investigate the utility of applying exome sequencing to monitor ctDNA levels. We evaluated two technologies that claim to enable sequencing of nano-gram levels of starting material without prior amplification. In brief, the data obtained through use of the ThruPLEX kit was superior, and therefore chosen for further evaluation. As we endeavored in this project to investigate the utility of ctDNA estimation through exome sequencing, we did not foresee the low level of ctDNA to be present in these samples. Only one sample contained detectable levels of ctDNA. Also, the levels of ctDNA was estimated to be one order of magnitude greater utilizing a break-point specific digital assay. It is probable that different mutations are found at varying fractions in the circulation as different clones in the heterogeneous primary tumor mass have different characteristics, a phenomenon noted previously [12], [30]. Still, our effort reveled several aspects not previously reported. First, the efficiency of sequence capture is low (<5%), impairing the use of exome sequencing to track specific mutations. The most probable reason is the library preparation in itself, as performing capture on eight samples simultaneously did not have a significant effect on coverage. Therefore, unless massive amounts of plasma is available, clinicians must resort to other methodologies to implement liquid biopsies as companion diagnostics, e.g. to search for KRAS mutations in colorectal patients treated with EGFR targeted therapy [31]. Nevertheless, to optimize the signal, we designed a new algorithm for the identification of PCR duplicates in merged read libraries. We envision this software to be broadly used, not only for cfDNA libraries, but also for formalin-fixed, paraffin embedded tumor material. Furthermore, by utilizing information in PCR duplicates to boost the quality of bases observed multiple times, the noise rate was significantly reduced to 1/35419, albeit at the cost of filtering out 39% of all data. Still, the sensitivity was limited to 1/2433, assuming the availability of 30 ng of cfDNA. Therefore, we investigated the sensitivity of performing “in silico” whole genome sequencing to 30× coverage, tracking 3000 mutations. As no enrichment is required for whole genome sequencing, data was only lost while reducing the noise rate. This lowered the sensitivity to 1/5747 and demarks the limitation to the optimized background noise rate in relation to the low fraction of ctDNA being present in clinical samples. Early-stage colorectal tumors was reported to harbored levels down to 1/10.000 [4]. At this fraction, 30× coverage would expect to give (30 fold coverage×3000 variants×0.61 percent kept after quality filtering×0.0001 fraction of ctDNA) 5.5 mutant reads by average. This must be put in the context of break-point specific PCR [5], [6], enabling the detection of single molecules. Assuming 10 ng of cfDNA and an unlikely 100% PCR efficiency, 3108 genome copies (10.000 pg×100%/3.218 pg per haploid human genome copy) would be available for interrogation, making it unlikely to detect such fractions without access to ≫10 ml of plasma, commonly not utilized in the literature. This also underlines the potential power to use multiple markers to track ctDNA. Nevertheless, to reach broader clinical implementation the challenges of using exome sequencing must be addressed. The low efficiency of sequence capture is likely to be improved with new library preparation approaches and in a system allowing for agitation during enrichment, an approach popular while in-house spotted arrays were utilized for gene expression experiments. The low fraction of ctDNA likely requires sampling of larger volumes of plasma, also for other technologies. Commonly 1–5 ml of plasma is used in ctDNA experiments, although 10 times as much could be obtained from patients without any obvious ethical dilemmas. For exome sequencing to be effective, using such input amounts, the inherent noise levels of short read data must be significantly reduced. A previously demonstrated approach to obliterate the background noise, was suggested by Loeb and colleagues is the addition of a random barcode to the Illumina adapter construct [29]. This enables the removal of basically all PCR related errors, but the introduction of a random barcode is likely to complicate adapter blocking during capture, with the risk of decreasing the already low efficiency of capture. Although we used plasma samples from cancer patients to estimate background noise, it is highly unlikely to have had an effect on our sensitivity estimates as the background noise rate was 16 times lower relatively unfiltered data, processed according to standard tools used by the academic community. Collectively, we demonstrate the use of exome sequencing as a tool to detect ctDNA but as explained, unless current inherent limitations of the approach are addressed, researchers and clinicians are going to have to resort to other options in order to do estimation of ctDNA in patients suffering from most organ-confined, low-grade primary cancers.

## Supporting Information

Figure S1 **An electropherogram from a BioAnalyzer instrument (Agilent) comparing the size-distribution of the simulated cell-free DNA (top) and a real plasma sample (bottom).** Y-axis, fluorescence units (FU). X-axis, fragment size in base pairs (bp).
(TIF)

Figure S2 **Definition of PCR duplicates.** The scheme describes how PCR duplicates are defined using analysis pipelines 1–3) in corresponding order. Left; Adapters are trimmed using SeqPrep. Subsequently, PCR duplicates are removed using MarkDuplicates (Picard), which takes the leftmost and the rightmost base into account for the identification of PCR duplicates. Middle; Reads not merged are processed as Left. PRC duplicates of merged reads are identified by leftmost starting position. Right; Reads not merged are processed as Left. PCR duplicates of merged reads are identified by starting positions and template length.
(EPS)

Figure S3 **Filtering somatic variants to obtain a set with minimum background noise.** Y-axis, number of variants from each sample. Variants were filtered vs. simple repeat regions, regions with low mappability, germline variants from the 1000 genomes project, de novo germline variants identified in these individuals. This set was used for detection of ctDNA. For clarity, PROT_EFF represents the number of variants with potential to affect protein function (non_synonymous, truncating etc.). Identification of regions harboring simple repeats and low mappability (50 mer) were downloaded from USCS genome browser. The 1000 genomes variant set was available in the GATK resource bundle.
(EPS)

Figure S4 **The evaluation was performed on a 5 Mb capture kit to facilitate sequencing the samples to saturation.** The variables evaluated are shown from left to right; 1) The number of samples captured simultaneously 2) The number of PCR cycles after capture but before sequencing. The Nimblegen SeqCap EZ standard protocol contains 18 rounds of PCR, yielding unnecessary high amounts of material. As amplification is performed on beads, it is not possible to use a qPCR instrument for the post-capture PCR 3) The Mondrian system and the ThruPLEX kit were evaluated for its capability to provide sequence libraries with high complexity for capture. 4) Input amounts of 1 ng and 10 ng representing cell-free DNA starting amounts commonly available from plasma samples. For both Mondrian and ThruPLEX, three independent library preparations were performed for both 1 ng and 10 ng of DNA all represented in the chart using 18 cycles of post-capture PCR. As 18 cycle post-capture PCR yielded micrograms of material, the impact was evaluated by taking the remaining material from the six ThruPLEX libraries and performing another round of capture. As the ThruPLEX data as superior we choose only to evaluate this variable using ThruPLEX libraries.
(EPS)

Figure S5 **Mean coverage obtained for the same sample using analysis pipelines 1)–3).** Identical samples are connected with lines. Left; 5 Mb target region used for technological evaluation. Right; Whole-exome data (26 Mb target region) obtained from plasma samples.
(EPS)

Figure S6 **The effect of varying input amounts of cell-free DNA for exome sequencing to tract ctDNA.** 1000 iterations were performed for each ctDNA fraction and input amount assessed here assuming 50 variants for each exome. The sensitivity is defined as the number of proportion of tests passing the significance threshold for each set of 1000 iteration (p<0.05, fishers' exact test, comparing the number of variant and reference reads from sample and background). Assuming 3 ng/ml the colored lines represent 1, 3, 10, 15 and 20 ml of plasma.
(EPS)

Table S1 An analysis of variance table showing the influence from different parameters on the library quality (measured as percent duplicated reads) when performing exome sequencing from small amounts of starting material. Listed parameters; cycles – 9 or 18 PCR cycles after capture but before sequencing; plex – indicates the number of samples captured simultaneously, here 1, 4 or 8; input – the starting amounts of DNA before library preparation, here 1 and 10 ng; prep – the technology used for library preparation, here Mondrian and ThruPLEX.
(PDF)

Table S2 The number of reads supporting either the mutations or reference bases in foreground- and background samples.
(PDF)
