# Supplementary figures and images for: Evaluation of Exome Sequencing to Estimate Tumor Burden in Plasma
Source: PLoS One. 2014 Aug 18;9(8):e104417. doi: 10.1371/journal.pone.0104417 (PMC4136786; doi:10.1371/journal.pone.0104417)

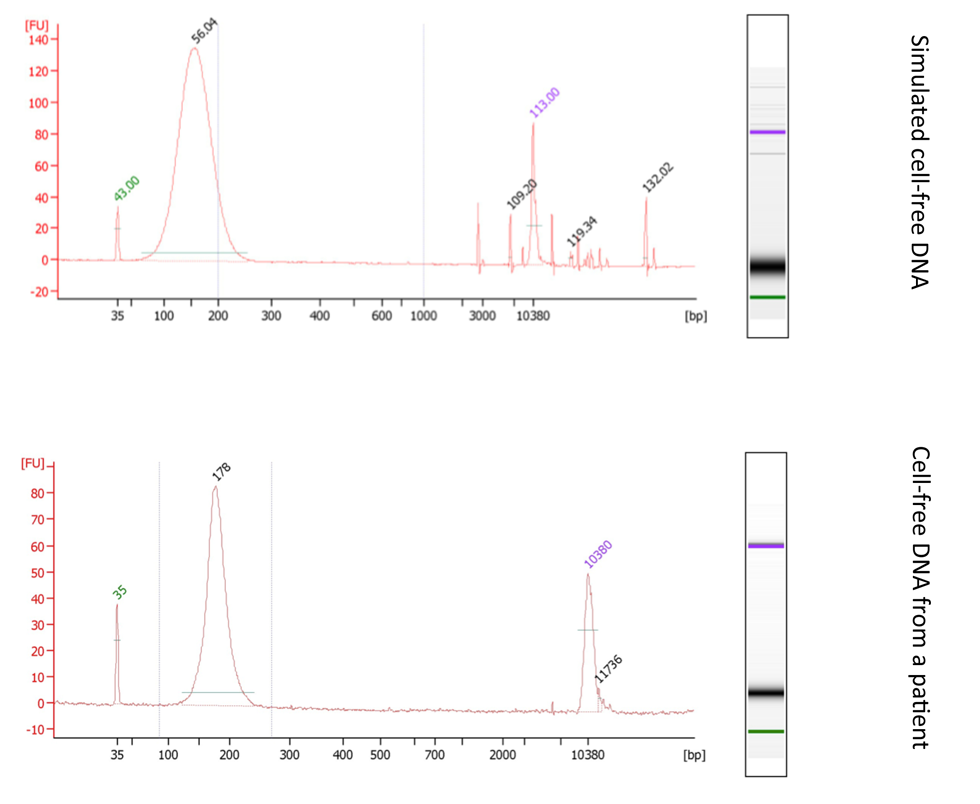

Supplement: Figure S1 — An electropherogram from a BioAnalyzer instrument (Agilent) comparing the size-distribution of the simulated cell-free DNA (top) and a real plasma sample (bottom). Y-axis, fluorescence units (FU). X-axis, fragment size in base pairs (bp). (TIF) [file pone.0104417.s001.tif]
