# Supplementary material for: Evaluation of Exome Sequencing to Estimate Tumor Burden in Plasma
Source: PLoS One. 2014 Aug 18;9(8):e104417. doi: 10.1371/journal.pone.0104417 (PMC4136786; doi:10.1371/journal.pone.0104417)
Supplement: Table S1 — An analysis of variance table showing the influence from different parameters on the library quality (measured as percent duplicated reads) when performing exome sequencing from small amounts of starting material. Listed parameters; cycles – 9 or 18 PCR cycles after capture but before sequencing; plex – indicates the number of samples captured simultaneously, here 1, 4 or 8; input – the starting amounts of DNA before library preparation, here 1 and 10 ng; prep – the technology used for library preparation, here Mondrian and ThruPLEX. (PDF) [file pone.0104417.s007.pdf]

Table S1 - Analysis of variance of parameters investigated during library prep technology evaluation.

```
Call:
lm(formula = PERCENT_DUPLICATION ~ cycles + plex + factor(amount) +
    factor(prep), data = temp)
```

Residuals:

Min	1Q	Median	3Q	Max
-0.163707	-0.058678	0.005087	0.067556	0.120113

Coefficients:

	Estimate	Std. Error	t-value	Pr(>|t|)	
(Intercept)	1.025502	0.120206	8.531	1.94e-06	***
cycles	0.001575	0.006518	0.242	0.813	
plex	0.005279	0.008109	0.651	0.527	
factor(amount)10	-0.277272	0.046164	-6.006	6.16e-05	***
factor(prep)T	-0.458509	0.057519	-7.972	3.90e-06	***

```
---
Signif. codes:  0 '***' 0.001 '**' 0.01 '*' 0.05 '.' 0.1 ' ' 1

Residual standard error: 0.09445 on 12 degrees of freedom
Multiple Adjusted R-squared: 0.8729
F-statistic: 28.47 on 4 and 12 DF,  p-value: 4.822e-06
```
