# Supplementary material for: Evaluation of Exome Sequencing to Estimate Tumor Burden in Plasma
Source: PLoS One. 2014 Aug 18;9(8):e104417. doi: 10.1371/journal.pone.0104417 (PMC4136786; doi:10.1371/journal.pone.0104417)
Supplement: Table S2 — The number of reads supporting either the mutations or reference bases in foreground- and background samples. (PDF) [file pone.0104417.s008.pdf]

Table S2 - Variants detected in plasma after optimal processing

Patient	Mut_plasma*	Ref_plasma*	Mut_plasma_bg**	Ref_plasma_bg**
SWE-54_B	1	1142	1	6866
SWE-54_A	0	1244	1	6866
BC_A	0	1235	0	6817
BC_B	0	2694	0	54841
BC_C	0	548	0	4059
BC_D	22	8681	3	70847
BC_E	0	644	0	4726
BC_F	0	2276	0	17934
BC_G	0	1127	1	10998

All plasma samples were collected before surgery, except SWE-54B, which was collected one month after surgery

* Reads containing mutant and reference bases in the corresponding patients' plasma sample

** Reads containing mutant and reference bases in all background samples
